# A systematic scoping review of latent class analysis applied to accelerometry-assessed physical activity and sedentary behavior

**DOI:** 10.1371/journal.pone.0283884

**Published:** 2024-01-22

**Authors:** Michael Kebede, Annie Green Howard, Yumeng Ren, Blake Anuskiewicz, Chongzhi Di, Melissa A. Troester, Kelly R. Evenson

**Affiliations:** 1 Department of Epidemiology, Gillings School of Global Public Health, University of North Carolina – Chapel Hill, Chapel Hill, North Carolina, United States of America; 2 Department of Biostatistics, Gillings School of Global Public Health, University of North Carolina – Chapel Hill, Chapel Hill, North Carolina, United States of America; 3 Carolina Population Center, University of North Carolina – Chapel Hill, Chapel Hill, North Carolina, United States of America; 4 Department of Biostatistics, University of California San Diego, San Diego, California, United States of America; 5 Division of Public Health Sciences, Fred Hutchinson Cancer Center, Seattle, Washington, United States of America; Linneaus University, SWEDEN

## Abstract

**Background:**

Latent class analysis (LCA) identifies distinct groups within a heterogeneous population, but its application to accelerometry-assessed physical activity and sedentary behavior has not been systematically explored. We conducted a systematic scoping review to describe the application of LCA to accelerometry.

**Methods:**

Comprehensive searches in PubMed, Web of Science, CINHAL, SPORTDiscus, and Embase identified studies published through December 31, 2021. Using Covidence, two researchers independently evaluated inclusion criteria and discrepancies were resolved by consensus. Studies with LCA applied to accelerometry or combined accelerometry/self-reported measures were selected. Data extracted included study characteristics and both accelerometry and LCA methods.

**Results:**

Of 2555 papers found, 66 full-text papers were screened, and 12 papers (11 cross-sectional, 1 cohort) from 8 unique studies were included. Study sample sizes ranged from 217–7931 (mean 2249, standard deviation 2780). Across 8 unique studies, latent class variables included measures of physical activity (100%) and sedentary behavior (75%). About two-thirds (63%) of the studies used accelerometry only and 38% combined accelerometry and self-report to derive latent classes. The accelerometer-based variables in the LCA model included measures by day of the week (38%), weekday vs. weekend (13%), weekly average (13%), dichotomized minutes/day (13%), sex specific z-scores (13%), and hour-by-hour (13%). The criteria to guide the selection of the final number of classes and model fit varied across studies, including Bayesian Information Criterion (63%), substantive knowledge (63%), entropy (50%), Akaike information criterion (50%), sample size (50%), Bootstrap likelihood ratio test (38%), and visual inspection (38%). The studies explored up to 5 (25%), 6 (38%), or 7+ (38%) classes, ending with 3 (50%), 4 (13%), or 5 (38%) final classes.

**Conclusions:**

This review explored the application of LCA to physical activity and sedentary behavior and identified areas of improvement for future studies leveraging LCA. LCA was used to identify unique groupings as a data reduction tool, to combine self-report and accelerometry, and to combine different physical activity intensities and sedentary behavior in one LCA model or separate models.

## Introduction

A growing number of epidemiologic studies use device-based measurements to quantify physical activity and sedentary behavior (e.g., physical behaviors), such as with accelerometers or activity trackers [1]. These devices can measure movement up to multiple times per second using raw data (e.g., 10–100 Hz) or summarize data into short windows of time called epochs (e.g., per minute or 15 seconds) [2]. Using epochs, authors classify the data into physical activity intensity levels (e.g., light, moderate, or vigorous) and sedentary behavior, and use monthly, weekly, or periodic sums to provide time in each category. While these approaches are effective in capturing multiple time frames, this grouping does not capture differences in patterns of accumulated physical activity and sedentary behavior over time [2]. The application of latent class analysis (LCA) to accelerometry can help elucidate patterns of physical activity and sedentary behavior over fixed time periods.

LCA is a measurement modeling technique that assumes the presence of underlying, unobserved, mutually exclusive categories which result in different patterns of variables observed in the data [3]. Using LCA, participant data on one or multiple characteristics can be partitioned into classes with other participants that share a similar pattern or behavior [4]. LCA can identify common classes derived through statistical modeling that otherwise might not have been discovered. This distinction has been described as a data-driven approach (i.e., using the data to identify and group people based on different patterns) in contrast to grouping people based on whether they meet certain criteria determined a priori [3]. LCA allows researchers to identify unique groups, which might not have been identified using a priori classifications, and to investigate how these unique groups vary in terms of participant characteristics or health outcomes.

LCA can be used to both identify and classify individuals into different subgroups that share similar patterns of physical activity and sedentary behaviors. One example is the use of LCA to identify the weekend warrior pattern of lower physical activity on weekdays and higher physical activity on weekends [5]. LCA can also be used to classify subgroups of individuals, based on differences in their patterns of physical activity and sedentary behavior data, and explore whether these subgroups differ by various correlates. LCA also incorporates many important aspects of physical activity and sedentary behavior, including frequency and time. The ability to identify behavioral patterns beyond cumulative time in order to maximize the variance explained is a notable advantage over traditional methods [6].

Multivariable analysis of variables used to fit the LCA model, such as accelerometry-assessed physical activity and sedentary behavior, is important in expanding the use of LCA for future research. To our knowledge, no published review discusses methods for examining accelerometry-assessed physical activity and sedentary behavior using LCA among both youths and adults. Therefore, we conducted a scoping review to systematically summarize how LCA has been applied to accelerometry-assessed physical activity and sedentary behavior in order to describe current research practice and identify methodologic gaps to aid future research using these methods.

## Methods

We conducted a systematic scoping review according to the Preferred Reporting Items for Systematic reviews and Meta-Analyses (PRISMA) Extension for Scoping Reviews [7]. The completed PRISMA checklist can be found in table S1 Appendix.

## Search strategy

We searched PubMed/Medline, Web of Science, CINHAL, SportDiscus, and Embase through December 31, 2021. The search terms used in these five databases can be found in table S2 Appendix.

The terms were entered based on previous review papers [8–10] as well as substantive experts from our team and searched using the advanced search field in each database. We downloaded results from each database as an RIS file type to import into Sciwheel, a reference management system. After duplicates were removed, search results from Sciwheel were transferred to Covidence for screening of titles/abstracts and full text. Reviewers were given the inclusion and exclusion criteria to aid in the screening and abstraction process. The screening and selection process were independently conducted between two researchers, with discrepancies resolved by consensus. A third reviewer provided a final check of all abstractions.

### Inclusion and exclusion criteria

To be included studies had to use English language and apply latent class methods to accelerometry for physical activity and/or sedentary behavior in an observational study. Studies that developed latent classes with both accelerometry and self-reported physical activity or sedentary behavior were included. We did not restrict on age or the behaviors (e.g., physical activity and sedentary behavior) as long as the measurements did not include sleep time or non-wear.

We excluded studies that derived latent classes with accelerometry using measures that were not physical activity or sedentary behavior. For example, studies which included diet combined with physical activity were excluded, since our focus was on physical behaviors only [11, 12]. Papers that did not use accelerometry or that used language other than English were excluded. Intervention studies were excluded from the review. To narrow the review specifically on LCA, we excluded (a) methods which focused more on polynomial regression-based modeling of patterns over time including growth mixed modeling and latent class growth modeling, (b) methods which focused on transitions between different latent variables over time including latent change score analysis and latent profile transition analysis, and (c) very broad methods including structural equation modeling and cluster analysis.

### Data extraction

The extraction form was developed by two reviewers as well as by consulting the quality assessment on LCA methods by Petersen et al. [9]. The extracted data were categorized into three sections: characteristics (e.g., study population, study design, race/ethnicity, country of study, age, gender, sample size, and data collection years), accelerometry data (e.g., brand/model, placement), and LCA model information (e.g., software, coding, model selection criteria, number of classes explored, final number of classes).

We conducted quality assessment of each study using a modified tool by Petersen et al. [9]. with additional questions we developed. Specific changes to the tool are detailed in table S3 Appendix. An answer of “yes” indicated higher quality.

## Results

### Descriptive characteristics of the included papers

**Fig 1:**
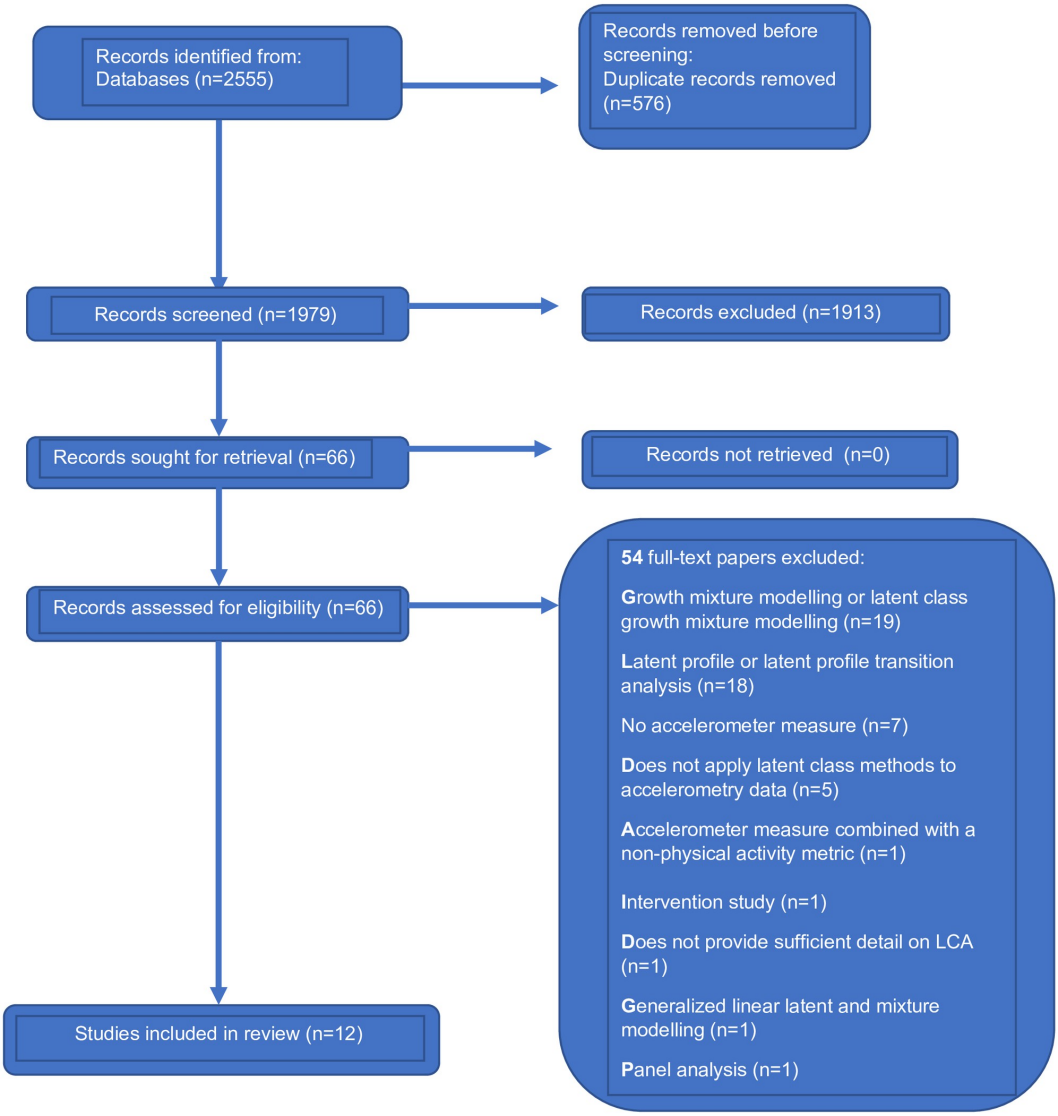
PRISMA flow diagram of the literature search and study selection process.

The literature search captured 2555 records from five databases. Sixty-six full-text papers were assessed for eligibility and 12 papers from 8 unique studies were included in the final analysis (Fig 1). Papers were included from a range of countries including the United States (n = 8) [2, 5, 6, 13–17], Australia (n = 2) [18, 19], the Netherlands (n = 1) [20], and Brazil (n = 1) [21] (Table 1). All included papers used cross-sectional study designs except one which was a prospective cohort study that collected accelerometry at one time point [2]. Among the 12 included papers, 5 used the adult National Health and Nutrition Examination Survey (NHANES) data [2, 5, 6, 14, 15], and 2 used the youth NHANES data [13, 17].

**Table 1:** Descriptive characteristics of included studies (N = 12 papers).

First Author (Year)	Study name	Data Collection Year(s)	Country of study	Study population	Analytic sample Size	Mean age (SD), range	N (%) Female	Race/Ethnicity N (%)
Metzger (2008) [14]	NHANES	2003–2004	US	≥ 20 years	3802	NR (NR),20–85	1956 (51.4)	Mexican 985 (19.5), Other Hispanic 152 (3.0), Non-Hispanic White 2689 (53.3), Non-Hispanic Black 994 (19.7), and Other/multiracial 221 (4.4)
Metzger (2010) [15]	NHANES	2003–2004	US	≥ 20 years	3458	NR (NR),20–85	NR, included both males and females	NR
Evenson (2015) [5]	NHANES	2003–2006	US	≥ 18 years	7931	NR (NR),18–85	4118 (51.9)	Non-Hispanic White 3953 (49.8), Non-Hispanic Black 1727 (21.8), Hispanic 1927 (24.3), and Other 324 (4.1)
Jones (2016) [6]	NHANES	2003–2006	US	≥ 20 years	7236	47 (NR), 20–85	3835 (53.0)	Hispanic 1696 (11.3), Non-Hispanic Black 1473 (10.7), Non-Hispanic White 3774 (72.7), and Other 293 (5.3)
Evenson (2017) [2]	NHANES Follow-up Study	2003–2011	US	≥ 40 years	4510	56.5 (NR), 40–85	2422 (53.7)	Non-Hispanic White 2489 (77.6), Non-Hispanic Black 927 (10.1), Hispanic 940 (7.9), and Other 154 (4.4)
Evenson (2016) [13]	NHANES	2003–2006	US	6–17 years	3998	NR (NR), 6–17	1992 (49.8)	NR
Jenkins (2017) [17]	NHANES	2003–2006	US	6–17 years	3984	12.2 (0.1), 6–17	2803 (48.8)	Non-Hispanic White 1469 (61.2), Non-Hispanic Black 1902 (14.8), Mexican American 1776 (12.4), and Other 460 (11.6)
Patnode (2011) [16]	Identifying Determinants of Eating and Activity in Adolescents Study; The Etiology of Childhood Obesity Study	IDEA:time 1 Nov 2006-May 2007; ECHO:time 1 Sep 2007-May 2008	Minneapolis/St. Paul, Minnesota, US	Children and adolescents from 6th-11th grade	720	14.7 (1.8), NR	368 (51.1)	White 609 (84.7)
Howie (2018) [19]	The Western Australia Pregnancy Cohort (Raine) Study	Estimated 2011–2013	Western Australia	Children of recruited expectant mothers between 1989 and 1991	628	22.1 (0.6), NR	324 (51.6)	NR
Jansen (2018) [20]	Physical Activity in Public Space Environments (PHASE) Study	2014	Rotterdam and Maastricht, the Netherlands	Randomly recruited residents aged 45–65 years, living in Rotterdam and Maastricht	222	56.8 (+/-6.1), 45–65	117 (52.7)	Autochthonous/indigenous 189 (85.1), Western immigrants 13 (5.9), Non-Western immigrants 17 (7.7), and NR 3 (1.3)
Parker (2019) [18]	The Neighborhood Activity in Youth Study	2014–2015	Melbourne, Australia	Secondary school students in Melbourne	473	15.0 (1.6), 12–18	277(58.6)	*Australian 457 (76.8)
Rocha de Faria (2020) [21]	NR	2018	Minas Gerais, Brazil	15 to 18 years old	217	16.1 (1.0), 15–18	107(49.3)	NR

Abbreviations: NR, Not reported; US, United States

Note that for NHANES studies, age is top coded such that the upper age in the dataset is 85 years.

*No race/ethnicity information was provided for participants other than “Australian”.

Data collection years for the studies ranged from 2003/2004 [15] to 2018 [21] and the sample size ranged from 217 [21]- 7931 [5] (Table 1). Ten of the 12 papers [2, 5, 6, 13–15, 17–19, 21] ensured that their sample was representative of their target population. For example, the NHANES data sampled the civilian, non-institutionalized US population [2, 5, 6, 13–15, 17]. The percentage of females in the studies ranged from 48.8% [17] to 58.6% [18].

### Accelerometry and latent class results for the included studies

Focusing on the 8 unique studies, 4 studies included youth (<18 years) participants [13, 16, 18, 21], with age ranging from 6 [13] to 17 [13] years. Four studies included adult participants [5, 14, 19, 20], with age ranging from 18 [5] to 65 [20] years. These eight studies measured physical activity and sedentary behavior using either combined accelerometer and self-report in the LCA (n = 3) [16, 18, 21] or accelerometry only (n = 5) [5, 13, 14, 19, 20] (Table 2). Studies used either the ActiGraph 7164 accelerometer (n = 4) [5, 13, 14, 16] or the ActiGraph GT3X+ (n = 4) [18–21] (S4 Appendix). The accelerometer was worn on the hip (n = 5) [5, 13, 14, 19, 21], waist (n = 1) [18], or was not reported (n = 2) [16, 20].

**Table 2:** Description of the accelerometer-related measures (N = 8 studies).

Accelerometer-related measures	N (%)	Reference
Physical activity measures for the LCA:		
Accelerometer only	5 (62.5)	[5, 13, 14, 19, 20]
Accelerometer and self-report	3 (37.5)	[16, 18, 21]
Sedentary behavior measures for the LCA:		
Yes	6 (75.0)	[5, 13, 16, 18, 19, 21]
No	2 (25.0)	[14, 20]
Accelerometer placement:		
Hip	5 (62.5)	[5, 13, 14, 19, 21]
Waist	1 (12.5)	[18]
Not reported	2 (25.0)	[16, 20]
Wear protocol:		
24 hours	2 (25.0)	[19, 21]
Waking hours	6 (75.0)	[5, 13, 14, 16, 18, 20]
Accelerometer epoch length:		
15 seconds	1 (12.5)	[21]
60 seconds	4 (50.0)	[5, 13, 14, 19]
Not reported	3 (37.5)	[16, 18, 20]
Accelerometer wear days:		
7 days	6 (75.0)	[5, 13, 14, 16, 19, 20]
8 days	2 (25.0)	[18, 21]
Number of hours for an adherent day:		
≥ 7 hours on weekends and ≥ 8 hours on weekdays	1 (12.5)	[18]
≥ 8 hours per week	4 (50.0)	[5, 13, 16]
≥ 10 hours per week	2 (25.0)	[19, 21]
≥ 360 epochs per hour from 9am to 9pm	1 (12.5)	[20]
Non wear time:		
≥ 20 minutes of consecutive zeros	1 (12.5)	[21]
≥ 30 minutes of consecutive zeros	1 (12.5)	[16]
≥ 60 minutes of consecutive zeros	2 (25.0)	[14, 18]
≥ 90 consecutive minutes of zero counts with allowance for up to 2 minutes of nonzero counts if no counts detected 30 minutes upstream or downstream from the interval (i.e., Choi algorithm [22])	2 (25.0)	[5, 13]
≥ 90 consecutive minutes of zero counts with allowance for up to 3 minutes of counts between 0 and 50	1 (12.5)	[19]
Not reported	1 (12.5)	[20]
Components of the LCA model:		
Physical activity and sedentary behavior in one model	4 (50.0)	[16, 18, 19, 21]
Physical activity and/or sedentary behavior in separate models	4 (50.0)	[5, 13, 14, 20]
Accelerometer coding for variables in LCA model:		
By day of week	3 (37.5)	[5, 13, 14]
By weekend vs weekday	1 (12.5)	[16]
Hour-by-hour	1 (12.5)	[20]
Weekly average	1 (12.5)	[21]
Dichotomized minutes per day	1 (12.5)	[18]
Sex-specific z-scores	1 (12.5)	[19]

Abbreviations: LCA, latent class analysis

Physical activity and sedentary behavior patterns were examined on various temporal scales including by day of week [5, 13, 14], weekend vs weekday [16], hour-by-hour [20], weekly average [21], dichotomized above or below the median number of minutes per day for the specific population [18], and derived sex-specific z-scores for physical activity and sedentary behavior variables [19]. Participants were instructed to wear accelerometers during waking hours (n = 6) [5, 13, 14, 16, 18, 20] or 24 hours (n = 2) [19, 21]. The number of accelerometer wear days reported was 7 days (n = 6) [5, 13, 14, 16, 19, 20] or 8 days (n = 2) [18, 21]. The recorded accelerometer epoch length varied from 15 seconds (n = 1) [21] to 60 seconds (n = 4) [5, 13, 14, 19] or was not reported (n = 3) [16, 18, 20]. Table S5 Appendix describes in detail the accelerometer derived variables and identified classes/cluster for all 12 unique studies.

The maximum number of classes considered ranged from up to 5 classes (n = 2) [18, 21] to up to 12 classes (n = 1) [5] (Table 3). However, despite the wide range explored, the number of final LCA classes was either 3 classes (n = 4) [16, 18, 20, 21], 4 classes (n = 1) [13], or 5 classes (n = 3) [5, 14, 19]. Researchers used a variety of criteria to guide the selection of the final of number of classes and model fit for model selection. The most common criteria to select the final best fitting model included the Bayesian Information Criteria (n = 5) [16, 18–21], substantive knowledge (n = 5) [5, 13, 14, 16, 19], sample size of classes (n = 4) [5, 13, 14, 18], and Akaike Information Criteria (n = 4) [16, 18, 19, 21]. Software used for the LCA analysis included Mplus (n = 4) [5, 13, 14, 18], Latent Gold (n = 2) [19, 20], SAS (n = 1) [16], specifically PROC LCA in SAS, and R software (poLCA package) (n = 1) [21].

**Table 3:** Description of the latent class analysis (N = 8 studies).

Characteristics	N (%)	Reference
Covariates adjusted in the LCA model:		
Yes	3 (37.5)	[14, 19, 21]
No or not reported	5 (62.5)	[5, 13, 16, 18, 20]
Number of classes explored:		
Up to 5	2 (25.0)	[18, 21]
Up to 6	3 (37.5)	[14, 16, 19]
Up to 7	1 (12.5)	[13]
Up to 9	1 (12.5)	[20]
Up to 12	1 (12.5)	[5]
Number of final classes explored:		
3	4 (50.0)	[16, 18, 20, 21]
4	1 (12.5)	[13]
5	3 (37.5)	[5, 14, 19]
Criteria to guide the selection of the final number of classes and model fit: *(not mutually exclusive categories)*		
Bayesian Information Criterion (BIC)	5 (62.5)	[16, 18–21]
Substantive knowledge, theoretical implication, conceptual meaning of the solution	5 (62.5)	[5, 13, 14, 16, 19]
Akaike information criterion (AIC)	4 (50.0)	[16, 18, 19, 21]
Sample size/class size	4 (50.0)	[5, 13, 14, 18]
Entropy	4 (50.0)	[14, 18, 19, 21]
Bootstrap likelihood ratio test (BLRT)	3 (37.5)	[5, 13, 14]
Visual inspection	3 (37.5)	[5, 13, 20]
G^2^ ~Likelihood ratio value	2 (25.0)	[16, 21]
Bootstrapped p-value using 500 replications for the log-likelihood difference between models	1 (12.5)	[19]
Classification errors	1 (12.5)	[19]
Odds of correct classification>5	1 (12.5)	[19]
Probabilities and proportions assigned to each class	1 (12.5)	[19]
Require each class comprise at least 15% of all observations	1 (12.5)	[20]
Interpretability of the item-response probabilities	1 (12.5)	[21]
Lo-Mendell-Rubin likelihood ratio test	1 (12.5)	[18]
Chi-squared goodness of fit	1 (12.5)	[21]
Average posterior probabilities >0.7	1 (12.5)	[19]
Number of unique physical activity and/or sedentary behavior variables in the model:		
1	4 (50.0)	[5, 13, 14, 20]
5	1 (12.5)	[21]
8	2 (25.0)	[18, 19]
12	1 (12.5)	[16]
Separate LCA performed by subgroup: *(not mutually exclusive categories)*		
By sex	3 (37.5)	[13, 16, 19]
By school characteristics (in school or out of school)	1 (12.5)	[13]
By age group	1 (12.5)	[13]
Software used for LCA:		
MPlus	4 (50.0)	[5, 13, 14, 18]
Latent Gold	2 (25.0)	[19, 20]
SAS	1 (12.5)	[16]
R	1 (12.5)	[21]

Abbreviations: LCA, latent class analysis

Three studies adjusted for covariates when developing their latent class models, including sociodemographic [14], health-related [21], and accelerometer measured variables (e.g., total number days of accelerometer) [19] (Table 3). Separate LCA subgroup analysis was performed by sex (n = 3) [13, 16, 19], by school characteristics (in school or out of school) (n = 1) [13], and by age (n = 1) [13]. In terms of specifying the benefits of LCA, 5 studies reported utilizing LCA to identify unique classes [5, 13, 14, 16, 19], 1 study reported LCA as a data reduction tool [18], and 4 studies reported using LCA to combine multiple physical activity variables [18–21].

In this review of 12 studies, several explored associations between LCA-derived variables with outcomes. Two youth studies explored the association between the physical activity and sedentary behavior latent classes to mental health [21] and physical health [21] outcomes, and cardiovascular disease risk factors [17]. Two adult studies explored associations between the LCA derived physical activity and sedentary behavior variables with all-cause mortality [2] and risk factors for metabolic syndrome [15].

### Quality assessment

The quality assessment tool, consisting of 16 questions, as applied to the 8 studies is provided in table S3 Appendix. Few studies reported their missing data mechanism (n = 3, question 1) and variables related to missingness (n = 2, question 2) (Table 4). Two studies considered covariates or the inclusion of the covariates into the LCA model (n = 2, question 7). Two studies included information about final replications (n = 2, question 9). None of the studies reported about the number of random start values (n = 0, question 8). Three studies made their supplementary files available (n = 3, question 16).

**Table 4:** Quality assessment tool assessing LCA in the included studies in publication date order (N = 8 studies).

Year	Author	Q1	Q2	Q3	Q4	Q5	Q6	Q7	Q8	Q9	Q10	Q11	Q12	Q13	Q14	Q15	Q16
2008	Metzger	N	Y	Y	Y	Y	Y	N	N	N	Y	Y	Y	Y	Y	Y	N
2011	Patnode	Y	N	Y	Y	Y	Y	NA	N	Y	Y	Y	Y	N	Y	Y	N
2015	Evenson	Y	N	Y	Y	Y	Y	NA	N	N	Y	Y	Y	N	Y	Y	Y
2016	Evenson	Y	Y	Y	Y	Y	Y	NA	N	N	Y	Y	Y	N	Y	Y	Y
2018	Howie	N	N	N	Y	Y	Y	Y	N	Y	Y	N	Y	Y	Y	Y	Y
2018	Jansen	N	N	N	N	Y	Y	NA	N	N	Y	N	Y	N	Y	Y	N
2019	Parker	N	N	Y	Y	Y	Y	NA	N	N	Y	N	Y	Y	Y	Y	N
2020	Rocha de Faria	N	N	N	Y	Y	Y	Y	N	N	Y	Y	Y	Y	Y	Y	N
Total “Yes”		3	2	5	6	8	8	2	0	2	8	5	8	4	8	8	3

Abbreviations: LCA, latent class analysis; N, no; NA, not applicable; Q, question; Y, yes

Note that Q1-Q16 are presented in S3 Appendix. Q1: Missing data reported, Q2: Variables related to missing data, Q3: Missing data in the analysis, Q4: Distribution of variables, Q5: Software mentioned, Q6: Coding of latent class variables, Q7: Covariates described, Q8: Random start values included, Q9: Iterations included, Q10: Model comparison using statistical tools, Q11: Number of fitted models reported, Q12: Participants per class reported, Q13: Entropy reported, Q14: Charts used, Q15: Final class solution described, Q16: Syntax files available

## Discussion

This systematic scoping review examined LCA modeling strategies using accelerometer-assessed physical activity and sedentary behavior. We modified and applied an existing quality assessment tool to provide important information on the quality and reporting of these LCA studies. We identified 12 papers from 8 unique studies using LCA methodology to accelerometry or combined accelerometry and self-report.

Our review focused on accelerometry-assessed physical activity and sedentary behavior using LCA, which provides important insight into how valuable LCA can be for this type of data. While studies in this review used different combinations of physical activity and sedentary behavior measures, including differences in cut points, all involved some degree of summarizing more detailed higher dimensional accelerometer-derived data using latent class analysis. This highlights one of the strengths of LCA, to identify physical activity variation across a wide range of movement profiles and summarize this into a smaller subset, in the case of LCA into the latent classes.

LCA used both physical activity and sedentary behavior data to generate classes [16]. Additionally, several studies found that classes differed by sex [13, 16, 19] and by age [13], thereby providing insights into correlates with LCA patterns over a particular time frame. One study [8] explored the use of LCA to identify patterns of physical activity and sedentary behaviors, and while this study did not focus exclusively on accelerometry-based data, they also found differences in the latent classes by sex, age, and school characteristics. DeFaria et al. [21] found that females with mental health disorders were more likely to belong to inactive classes. Metzger et al. [15] reported that metabolic syndrome risk factors were lower among classes with more moderate-to-vigorous physical activity.

Using these two examples, identifying specific classes might provide important insight into interventions addressing these outcomes.

Understanding class membership is important in order to develop interventions that can be tailored to groups. Including individual covariates (e.g., sociodemographic) in LCA models to help predict class membership has been shown to improve model interpretation, identification, and fit [23]. Wurpts et al. [23] suggests that the use of a larger number of high-quality variables and the inclusion of at least one covariate under a strong theoretical basis positively affect LCA model estimation, and that these factors can sometimes compensate for other suboptimal conditions (e.g., a relatively small sample size). In our review, about half of the unique studies reported including covariates in the model.

Almost all studies in this review listed their criteria to guide the selection of the final number of classes and models fit. The most common criteria used to test the relative fit of models were BIC, AIC, BLRT, substantive knowledge including practical interpretation of what each class represented, visual inspection (to ensure classes were sufficiently separated from each other or large enough to be of the researcher’s interest), and entropy. Multiple criteria in the selection of final classes and model fit such as large sample sizes, AIC, BIC, and entropy are recommended to detect minor differences in class samples [8]. Entropy is frequently used in studies to quantify how well participants are classified into latent classes and to quantify the quality of classification and separation between groups in a log based model [24]. Hence, we included entropy in our quality assessment tool to evaluate its adoption in the reviewed studies.

The quality assessment tool identified areas that generally needed further clarification such as variable missingness, description of covariates (if used), number of final replications/iterations used, and total number of fitted models reported. A majority of the studies clearly stated the study objective in relation to preferences and described methods and procedures. LCA models assume data are missing at random and missing data on a covariate or grouping variable can present a complex issue, therefore, confirming this assumption prior to the analysis is important [3]. As the application of LCA in physical activity and sedentary behavior expands, investigators should improve the quality and reporting of LCA studies. This can be achieved by addressing missing data in the LCA and explain how and where the missingness is attributed. Moreover, future studies should describe model selection criteria, the final number of classes which will allocate sufficient sample size in each class, and covariates included in the model. Utilizing an appropriate checklist when writing the methods and presenting the results. For this study we expanded upon an existing checklist for LCA [9] and a checklist for latent trajectory studies is available elsewhere [25].

### Strengths and limitations

The strengths of this review include extensive search using five databases and the application of a quality assessment tool adapted for this review. Despite these strengths, several limitations exist. First, we restricted the review to include papers published in the English language only and missed potential studies in other languages. We also focused on peer-reviewed studies and may have missed additional studies in the grey literature. Second, we limited our methods to only LCA, and therefore did not include other studies which used latent variables to summarize physical activity and sedentary behaviors (i.e., growth mixed modeling, latent class growth analysis, latent change score analysis, latent profile transition analysis, factor analysis, or cluster analysis) or alternative clustering algorithms (i.e. k-means clustering). However, we narrowed the focus so as to better understand LCA specifically applied to accelerometry. Third, while we did not include studies which focused on patterns of accelerometry data collected across multiple visits, it was due to the fact that in these cases alternative methods were used which may require guidance different or in addition to that required by LCA. Our focus on LCA methods specifically allowed us to provide guidance for this specific type of analysis.

## Conclusions

The aim of this scoping review was to describe methods and procedures used in applying LCA to accelerometry. Across a variety of study populations, LCA identified unique subgroups of physical activity and sedentary behavior, and these unique classes were associated with both health outcomes and individual characteristics. LCA was used to identify unique groupings as a data reduction tool, to combine self-report and accelerometry, and to combine different physical activity intensities and sedentary behavior in one LCA model or separate models. This review provided insight into the reporting of LCA in accelerometry studies and identified areas of improvement for future studies leveraging LCA.

## Supporting information

S1 Appendix Preferred Reporting Items for Systematic reviews and Meta-Analyses extension for Scoping Reviews (PRISMA-ScR) checklist. (PDF)

S2 Appendix Search terms by databases. (PDF)

S3 Appendix Quality assessment tool adapted from Petersen et al. [9]. (PDF)

S4 Appendix Description of accelerometer and self-reported measures used in the LCA for physical activity and sedentary behavior (N = 8 studies). (PDF)

S5 Appendix Description of accelerometer derived variables and possible outcomes (N = 12 studies). (PDF)
