# Supplementary material for: A systematic scoping review of latent class analysis applied to accelerometry-assessed physical activity and sedentary behavior
Source: PLoS One. 2024 Jan 22;19(1):e0283884. doi: 10.1371/journal.pone.0283884 (PMC10802947; doi:10.1371/journal.pone.0283884)
Supplement: S2 Appendix — (PDF) [file pone.0283884.s002.pdf]

S2 Appendix: Search terms by databases

Database	Search terms	Filters
PubMed/Medline	"Latent Class Analysis"[Mesh] OR "Latent Class Analys*" OR "Latent Variable Model*" OR "Latent Class Model*" OR "LCA" OR "person oriented analys*" OR "latent class" OR "Latent class transition model" AND "Aerobic endurance"[tiab] OR "Aerobic activities"[tiab] OR "Aerobic activity"[tiab] OR Bicycl*[tiab] OR "Cardiovascular activities"[tiab] OR "Cardiovascular activity"[tiab] OR "Endurance activities"[tiab] OR "Endurance activity"[tiab] OR "Endurance training"[tiab] OR Exercise[mh] OR Exercise*[tiab] OR "Free living activities"[tiab] OR "Free living activity"[tiab] OR "Leisure-time physical activity"[tiab] OR "Lifestyle activities"[tiab] OR "Lifestyle activity"[tiab] OR "Physical activity"[tiab] OR "Physical activities"[tiab] OR "Physical conditioning"[tiab] OR "Recreational activities"[tiab] OR "Recreational activity"[tiab] OR Walking[tiab] OR "Sedentary lifestyle"[mh] OR "Sedentary behavior"[MeSH Terms] OR "Sedentary behavior"[All Fields] OR Sedentary[tiab] OR physical activity [Text Word] OR acceleromet*[tiab] OR "fitness track*"[tiab] OR "activity monitor*"[tiab] or "motion sens*"[tiab] or "device-based"[tiab] or "activity tracker*"[tiab] OR "accelerometer"[tiab]	Limit to English Language, no animal studies, an no editorials, reviews, commentaries, letters, conference abstracts
EMBASE	('physical activity'/exp OR 'aerobic exercise'/exp OR 'cardiovascular activity'/exp OR 'endurance training'/exp OR 'exercise'/exp OR 'leisure time physical activity'/exp OR 'sedentary lifestyle'/exp OR 'accelerometer'/exp OR 'activity monitor'/exp OR 'activity tracker'/exp OR 'motion sensor'/exp) AND ('latent class analysis'/exp OR 'latent variable model'/exp OR 'latent class model'/exp OR 'mixture model'/exp OR 'latent class growth analysis'/exp)	Limit to English Language, no animal studies, and no editorials, reviews, commentaries, letters, conference abstracts
SportDiscus	(("PHYSICAL activity") OR ("Physical training & conditioning" OR "aerobic exercises") OR ("Exercise") OR ("sedentary lifestyles") OR ("Accelerometers")) AND ("Latent Class Analysis" OR "Latent Class Analys*" OR "person oriented analys*" OR "latent class" OR "Latent class transition model" OR "Latent class growth model")	Limit to English Language, no animal studies, and no editorials, reviews, commentaries, letters, conference abstracts

Web of Science	(((((((((((((((ALL=(Physical activity)) OR ALL=(Sedentary lifestyle)) OR ALL=(Sedentary behavior)) OR ALL=(Aerobic endurance)) OR ALL=(Aerobic activities)) OR ALL=(endurance activities)) OR ALL=(leisure time physical activity)) OR ALL=(acceleromet*)) OR ALL=(activity monitor*)) OR ALL=(activity tracker)) OR ALL=(physical activities))<br>AND<br>ALL=(Latent class analysis)) OR ALL=(Latent variable model)) OR ALL=(latent class model)) OR ALL=(person oriented analys*)) OR ALL=(latent class growth model)) OR ALL=(latent class transition model)	Limit to English Language, no animal studies, and no editorials, reviews, commentaries, letters, conference abstracts
CINHAL	("Aerobic endurance" OR "Aerobic activities" OR "Aerobic activity" OR "Bicycl*" OR "Cardiovascular activities" OR "Cardiovascular activity" OR "Endurance activities" OR "Endurance activity" OR "Endurance training" OR "Exercise" OR "Exercise*" OR "Free living activities" OR "Free living activity" OR "Leisure-time physical activity" OR "Lifestyle activities" OR "Lifestyle activity" OR "Physical activity" OR "Physical activities" OR "Physical conditioning" OR "Recreational activities" OR "Recreational activity" OR "Walking" OR "Sedentary lifestyle" OR "Sedentary behavior" OR "Sedentary" OR "acceleromet*" OR “fitness track*” OR “activity monitor*” or “motion sens*” or “device-based” OR “activity tracker*” OR “accelerometer”)<br>AND<br>("Latent Class Analysis" OR "Latent Class Analys*" OR "Latent Variable Model*" OR "Latent Model*" OR “person oriented analys*” OR “latent Class class” OR "Latent class*" OR “Latent class transition model” OR "Latent class transition analysis")	Limit to English Language, no animal studies, and no editorials, reviews, commentaries, letters, conference abstracts
