# Supplementary material for: A systematic scoping review of latent class analysis applied to accelerometry-assessed physical activity and sedentary behavior
Source: PLoS One. 2024 Jan 22;19(1):e0283884. doi: 10.1371/journal.pone.0283884 (PMC10802947; doi:10.1371/journal.pone.0283884)
Supplement: S3 Appendix — (PDF) [file pone.0283884.s003.pdf]

S3 Appendix: Quality assessment tool adapted from Petersen et al. [9]

**Petersen et al. (2019) tool**	**Adaptation and clarification for this review**	**Question Number**
Is the missing data mechanism reported?	Is the missing data mechanism reported?	1
Is a description provided of what variables are related to attrition/ missing data?	Is a description provided of what variables are related to attrition/ missing data?	2
Is a description provided of how missing data in the analyses were dealt with?	Is a description provided of how missing data in the analyses were dealt with?	3
Is information about the distribution of the observed variables included? e.g., continuous, ordinal, count, zero inflated,	Is information about the distribution of the observed variables included? e.g., continuous, ordinal, count, zero inflated	4
Is the software mentioned?	Is the software mentioned?	5
Are parameter restrictions reported?	Was sufficient detail provide about how the latent class variables were coded?	6
If covariates have been used, can analyses still be replicated? i.e., is sufficient detail provided about the method used to include covariates?	If covariates have been used, was sufficient detail provided about how the covariates were coded and how they were included in the LCA? Answers: no covariates, no, yes	7
Is information reported about the number of random start values and final iterations included?	Is information reported about the number of random start values included?	8
	Is information about the number of final iterations/ replications included?	9
Are the model comparison (and selection) tools described from a statistical perspective?	Were statistical tools (e.g., entropy) used for model comparison and selection?	10
Are the total number of fitted models reported, including a one-class solution?	Are the total number of fitted models reported?	11
Are the number of cases per class reported for each model (absolute sample size, or proportion)?	Are the number of participants per class reported for the final model (absolute sample size, or proportion)?	12

Is entropy reported?	Is entropy reported?	13
Are plots/bar charts included with the response patterns of the classes/profiles for each model?	Are plots/bar charts included with the response patterns of the classes/profiles for the final model?	14
Are characteristics of the final class solution numerically described? (i.e., means, SD/SE, n, CI, etc.)	Are characteristics of the final class solution numerically described? (i.e., means, standard deviation, standard error, sample size, confidence intervals, etc.)	15
Are the syntax files available (either in the appendix, supplementary materials, or from the authors)?	Are the syntax files available (either in the appendix, supplementary materials, or from the authors)?	16
