# Supplementary material for: A systematic scoping review of latent class analysis applied to accelerometry-assessed physical activity and sedentary behavior
Source: PLoS One. 2024 Jan 22;19(1):e0283884. doi: 10.1371/journal.pone.0283884 (PMC10802947; doi:10.1371/journal.pone.0283884)
Supplement: S4 Appendix — (PDF) [file pone.0283884.s004.pdf]

S4 Appendix: Description of accelerometer and self-reported measures used in the LCA for physical activity and sedentary behavior (N=8 studies)

**Author Last Name (Year)**	**Primary measures in the LCA**	**Accelerometry Used**	**Accelerometer Placement**	**Wear protocol**	**Accelerometer Wear (# days)**	**# hours for adherence**	**Accelerometer Epoch Length**	**Distribution method of accelerometer**
Metzger (2008) [14]	Accelerometer only	ActiGraph 7164	Hip	Waking hours	7	≥ 8 hours/day	1 minute	In person
Patnode (2011) [16]	Accelerometer and self-report	ActiGraph 7164	Not reported	Waking hours	7	≥ 8 hours/day	Not reported	In person
Evenson (2015) [5]	Accelerometer only	ActiGraph 7164	Hip	Waking hours	7	≥ 8 hours/day	1 minute	In person
Evenson (2016) [13]	Accelerometer only	ActiGraph 7164	Hip	Waking hours	7	≥ 8 hours/day	1 minute	In person
Howie (2018) [19]	Accelerometer only	ActiGraph GT3X+	Right hip	24 hour	7	≥ 10 hours/day	60 seconds	In person
Jansen (2018) [20]	Accelerometer only	ActiGraph GT3X+	Not reported	Waking hours	7	≥ 360 epochs/ hour between 9am-9pm	Not reported	Not reported
Parker (2019) [18]	Accelerometer and Self-report	ActiGraph GT3X+	Waist	Waking hours	8	≥ 8 hours on weekdays and >=7 hours on weekend day	Not reported	In person
Rocha de Faria (2020) [21]	Accelerometer and Self-report	ActiGraph GT3X	Right hip	24 hour	8	≥ 10 hours/day	15 seconds	In person
