# Supplementary material for: A systematic scoping review of latent class analysis applied to accelerometry-assessed physical activity and sedentary behavior
Source: PLoS One. 2024 Jan 22;19(1):e0283884. doi: 10.1371/journal.pone.0283884 (PMC10802947; doi:10.1371/journal.pone.0283884)
Supplement: S5 Appendix — (PDF) [file pone.0283884.s005.pdf]

S5 Appendix: Description of accelerometer derived variables and possible outcomes (N=12)

**First Author (Year)**	Accelerometer-derived variables	Identified classes/clusters
Metzger (2008)[14]	1. MVPA minutes<br>2. MVPA bout minutes<br>3. VPA minutes	1. MVPA minutes, latent classes class 1 (least active) – class 5 (most active)<br>2. MVPA bout minutes, latent classes class 1 (least active) – class 5 (most active)<br>3. VPA minutes, model failed to produce results because of small class sizes
Metzger (2010)[15]	1. MVPA minutes	1. MVPA minutes, latent classes class 1 (least active) – class 5 (most active)
Evenson (2015)[5]	1. Counts/minute per day<br>2. Percent of MVPA out of total wearing time per day<br>3. Percent of MVPA bouts out of total wearing time per day<br>4. Percent of sedentary behavior out of total wearing time per day<br>5. Percent of sedentary bouts out of total wearing time per day	1. Average counts per minute, latent classes class 1 (least active) – class 6 (most active)<br>2. Percent of MVPA out of total wearing time per day, latent classes class 1 (least active) – class 5 (most active)<br>3. Percent of MVPA bouts out of total wearing time per day, latent classes class 1 (least active) – class 5 (most active)<br>4. Percent of sedentary behavior out of total wearing time per day, latent classes class 1 (most sedentary) – class 5 (least sedentary)<br>5. Percent of sedentary bouts out of total wearing time per day, latent classes 1(Most

		sedentary) – 7(least sedentary)
Jones (2016)[6]	1. Average counts per minute<br>2. Percent MVPA (>=2020 counts/min)<br>3. Percent MVPA (>=760 counts/min)<br>4. Percent sedentary behavior	1. Average counts per minute, latent classes class 1 (least active) – class 6 (most active)<br>2. Percent MVPA (>=2020 counts/min), latent classes class 1 (least active) – class 5 (most active)<br>3. Percent MVPA (>=760 counts/min), latent classes class 1 (least active) – class 6 (most active)<br>4. Percent sedentary behavior, latent classes class 1 (most sedentary) – class 6 (least sedentary)
Evenson (2017)[2]	1. Counts/minute per day<br>2. Percentage of MVPA/ MVPA_lifestyle out of total wear time/day<br>3. Percentage of MVPA/MVPA_lifestyle bouts out of total wear time/day<br>4. Percentage of sedentary behavior out of total wear time/day<br>5. Percentage of sedentary bouts out of total wear time/day	1. Counts/minute per day, latent classes 1(least active) – 6 (most active)<br>2. Percentage of MVPA per day, latent classes 1(least active) – 5 (most active)<br>3. Percentage of MVPA bouts per day, latent classes 1(least active) – 5 (most active)<br>4. Percentage of MVPA_lifestyle per day, latent classes 1(least active) – 6 (most active)<br>5. Percentage of MVPA_lifestyle bouts per day, latent classes 1(least active) – 5 (most active)<br>6. Percent of sedentary behavior per day, latent classes 1(most sedentary) – 5 (least sedentary)<br>Percentage of sedentary bouts per day, latent classes 1(Most

		sedentary) – 7(least sedentary)
Evenson (2016)[13]	1. Counts per minute per day<br>2. Percent of sedentary behavior out of total wearing time per day<br>3. Percent of light activity out of total wearing time per day<br>4. Percent of MVPA out of total wearing time per day<br>5. Percent of vigorous activity out of total wearing time per day	1. Counts per minute per day, latent classes class 1 (least active) – class 4 (most active)<br>2. Percent of sedentary behavior out of total wearing time per day, latent classes class 1 (most sedentary) – class 4 (least sedentary)<br>3. Percent of light activity out of total wearing time per day, latent classes class 1 (least light) – class 4 (most light)<br>4. Percent of MVPA out of total wearing time per day, latent classes class 1 (least MVPA) – class 4 (most MVPA)<br>5. Percent of vigorous activity out of total wearing time per day, latent classes class 1 (least Vigorous) – class 4 (most Vigorous)
Jenkins (2017) [17]	1. Percent MVPA per day<br>2. Percent sedentary behavior per day<br>3. Counts per minute	1. Percent MVPA per day, latent classes class 1 (least active) - class 4 (most active)<br>2. Percent sedentary behavior per day, latent classes class 1 (most sedentary) – class 4 (least sedentary)<br>3. Counts per minute, latent classes class 1 (least active) – class 4 (most active)

Patnode (2011)[16]	1. MVPA-weekdays, ≥30 min/day<br>2. MVPA-weekend days, ≥30 min/day	Item response probabilities were generated for the accelerometer derived variables with 3 classes (Class 1-3)
Howie (2018)[19]	1. Steps/day<br>2. MVPA (min/day)<br>3. MVPA SD (min/day)<br>4. MVPA intensity (mean counts)<br>5. MVPA duration (mean min/bout)<br>6. Sedentary-to-light ratio<br>7. Sedentary-to-light SD<br>8. Sedentary bouts (mean min/bout)	All accelerometer derived variables were classified by activity phenotypes ranging from Activity phenotype 1 – Activity phenotype 5
Jansen (2018)[20]	1. Percentages of LPA<br>2. Percentage of MVPA	1. Percentages of LPA, (Day type 1- Day type 4)<br>2. Percentages of MVPA, (Day type 1- Day type 4)
Parker (2019)[18]	1. Sedentary time duration<br>2. MVPA (mins/day)	Three typologies were identified.<br>Typology 1 (physically inactive, highly sedentary), Typology 2 (moderately active, high screen-time), Typology 3 (highly active, low sedentary)
Rocha de Faria (2020)[21]	1. MVPA<br>2. Sedentary behavior<br>3. Light physical activity<br>4. Number of steps	After interpretation of item response probabilities 3 classes were generated. Class 1 (active-non sedentary), class 2 (inactive non sedentary), class 3 (inactive sedentary)

Abbreviations: MVPA, moderate to vigorous physical activity; SD, standard deviation
